# The Positive Impact of Early Frailty Levels on Mortality in Elderly Patients with Severe Aortic Stenosis Undergoing Transcatheter/Surgical Aortic Valve Replacement

**DOI:** 10.3390/jcdd10050212

**Published:** 2023-05-13

**Authors:** Annamaria Mazzone, Serena Del Turco, Giuseppe Trianni, Paola Quadrelli, Marco Marotta, Luca Bastiani, Tommaso Gasbarri, Andreina D’Agostino, Massimiliano Mariani, Giuseppina Basta, Ilenia Foffa, Silverio Sbrana, Cristina Vassalle, Marcello Ravani, Marco Solinas, Sergio Berti

**Affiliations:** 1 Diagnostic and Interventional Cardiology Department, Fondazione Toscana Gabriele Monasterio, 54100 Massa, Italya.dagostino@monasterio.it (A.D.); massimiliano.mariani@ftgm.it (M.M.); sergio.berti@ftgm.it (S.B.); 2 CNR Institute of Clinical Physiology, 56124 Pisa, Italy; 3 Adult Cardiosurgery Department, Fondazione Toscana Gabriele Monasterio, 54100 Massa, Italy; paola.quadrelli@ftgm.it (P.Q.); tommaso.gasbarri@ftgm.it (T.G.);; 4 CNR Institute of Clinical Physiology, 54100 Massa, Italy; 5 Fondazione Toscana Gabriele Monasterio, 56124 Pisa, Italy; cristina.vassalle@ftgm.it

**Keywords:** aortic stenosis, pre-frailty, frailty, transcatheter aortic valve replacement, mortality

## Abstract

**Background:** Frailty is highly common in older patients (pts) undergoing transcatheter aortic valve replacement (TAVR), and it is associated with poor outcomes. The selection of patients who can benefit from this procedure is necessary and challenging. The aim of the present study is to evaluate outcomes in older severe aortic valve stenosis (AS) pts, selected by a multidisciplinary approach for surgical, clinical, and geriatric risk and referred to treatment, according to frailty levels. **Methods:** A total of 109 pts (83 ± 5 years; females, 68%) with AS were classified by Fried’s score in pre-frail, early frail, and frail and underwent surgical aortic valve replacement SAVR/TAVR, balloon aortic valvuloplasty, or medical therapy. We evaluated geriatric, clinical, and surgical features and detected periprocedural complications. The outcome was all-cause mortality. **Results:** Increasing frailty was associated with the worst clinical, surgical, geriatric conditions. By using Kaplan–Meier analysis, the survival rate was higher in pre-frail and TAVR groups (*p* < 0.001) (median follow-up = 20 months). By using the Cox regression model, frailty (*p* = 0.004), heart failure (*p* = 0.007), EF% (*p* = 0.043), albumin (*p* = 0.018) were associated with all-cause mortality. **Conclusions:** According to tailored frailty management, elderly AS pts with early frailty levels seem to be the most suitable candidates for TAVR/SAVR for positive outcomes because advanced frailty would make each treatment futile or palliative.

## 1. Introduction

Aortic stenosis (AS) is the most common valvular disease associated with aging and it is on the rise in Western countries [1]. Current definitive interventions include surgical aortic valve replacement (SAVR) or transcatheter aortic valve replacement (TAVR) [1,2]. TAVR is a consolidated, innovative treatment for patients previously untreated due to high or prohibitive surgical risk [3]. Randomized trials have indeed demonstrated improvements in survival and symptoms after TAVR compared to medical therapy (MT) [3,4]; however, there is still a percentage of treated patients who do not benefit from TAVR, either due to subsequent death or a worsening of their quality of life [3,4,5]. Hence, current guidelines define futility as a lack of survival or worsening of quality of life at 1-year post-TAVR, discouraging the intervention. In these cases, invasive treatment may be a dangerous risk exposure for the patient and a misuse of health care resources [6]. These critical issues highlight the importance of establishing preliminary criteria to avoid inappropriate interventions in elderly patients [7].

Frailty is a geriatric syndrome characterized by reduced physiological reserve and increased vulnerability to adverse events that occur in about 25–50% of elderly patients with cardiovascular disease [8,9]. Frailty develops through a continuum, starting from a physiologically robust and independent condition to disability and dependence or, even worse, hospitalization and death [10]. In addition to clinical and surgical risk factor stratification, the multidisciplinary Heart Team needs to weigh factors, such as frailty, multimorbidity, and disability, to assess the predicted benefit of TAVR in the elderly population [7,11]. Indeed, pre-interventional assessment of frailty in association with the common traditional risk scores is useful for post-interventional prognosis [12,13]. By using physical, multidimensional frailty, or single markers, several studies showed that pre-interventional frailty is an independent predictor of mortality. [12,13,14,15]; thus, a comprehensive frailty assessment prior to TAVR could be a strategic approach in clinical decision making for the tailored management of patients with AS, an approach that could improve patient outcomes. The quantification of clinical features and functional levels may be useful in measuring reversible conditions in which TAVR could improve patient outcomes [14,16,17,18].

This pilot study aimed to implement an open, multidisciplinary pre-operative path dedicated to symptomatic AS elderly patients, characterized by an integrated clinical, surgical, and geriatric risk assessment for “frailty-based management” and tailored treatment of valve disease. Thus, according to the different frailty levels, patients underwent SAVR, TAVR, balloon aortic valvuloplasty (BAV), or medical therapy (MT), and the middle-time mortality was evaluated.

## 2. Materials and Methods

### 2.1. Study Population

Our study is a single-center prospective study conducted at Ospedale del Cuore FTGM, in Massa, Italy. We implemented a pilot clinical project for elderly patients with severe AS to optimize the TAVR pathway. A multidisciplinary Heart Team, identified among FTGM healthcare professionals from different fields working in close coordination and organization (clinical/geriatric cardiologist, interventional cardiologist, radiologist, cardiac surgeon, anesthetist, psychologist, therapist), activated an outdoor pre-TAVR pathway to detect surgical, cardiovascular, and geriatric risk in order to refer patients to the tailored treatment of AS (SAVR, TAVR, BAV, MT). The population consisted of patients >65 years (n = 109), randomly recruited from March 1, 2016, to March 30, 2020, among elderly symptomatic AS patients for multidisciplinary assessment. AS with an effective orifice area <1 cm^2^ and/or <0.6 cm^2^/m^2^ body surface area was considered severe [19]. All the included subjects had cardiac symptoms of advanced AS (17), while those patients with acute heart failure, hemodynamic shock were excluded at the moment of clinical evaluation of day service. The study was approved by the local Ethics Committee (no. 22239), and patients provided informed written consent.

### 2.2. Clinical/Interventional Cardiovascular Assessment

Patients were interviewed for New York Heart Association (NYHA) classification, cardiovascular risk factors, and prior cardiovascular events, including last acute heart failure, polypharmacotherapy, and physical examination. Moreover, 12 derivation ECGs and peripheral venous routine laboratory samples were performed. We used transthoracic echocardiography imaging to evaluate left ventricular ejection fraction, transvalvular mean gradient, mitral regurgitation, and pulmonary artery pressure [19]. Patients with creatinine <1.4 mg/dL also underwent aortic computed tomography to evaluate aortic root, vascular access site specifications, and arterial calcification for TAVR. Surgical and anesthetist evaluation was performed by Society of Thoracic Surgeons (STS) Predicted Risk of Mortality (STS-PROM) score [20] (score was dichotomized at standard cut-off points: STS score ≥ 4% high risk versus <4% lower risk) for anatomical and functional features [13].

### 2.3. Fried Phenotypic Score and Multidimensional Geriatric Assessment (MGA)

According to the comprehensive model, the identification of frailty can be detected by phenotypic Fried criteria (i.e., robust = 0, pre-frail = 1–2, early frail = 3, frail = 4–5) [21]. We also considered the following parameters: *weight loss* > 4.5 kg (10 lbs), lost unintentionally in the prior year; *weakness*, using hand grip <5.85 kg (12.89 lbs) for males, <3.37 kg (7.43 lbs) for females; *exhaustion*, self-reported at least 3 days/week; *slowness*, using timed up-and-go test (TUG) for gait function, TUG at ≥ 20 s (mobility impairment) versus <20 s (normal gait function) [22]; *low activity*, using the Physical Activity Scale for the Elderly (PASE) [23]. Moreover, we included multidimensional geriatric assessment (MGA) to evaluate comorbidities, disability, cognitive function, depression, and nutritional status using the following validated indices. The indices used in this study were (1) Charlson Index (CI) with a cut-off value of >2 for comorbidity [24]; (2) basic activities of daily living (BADL) and instrumental activities of daily living (IADL) for disability [25,26]; (3) mini–mental state examination for cognitive function evaluation (MMSE at≤ 18 points, cognitive impairment) [27]; (4) Geriatric Depression Score for mood disorder (GDS at ≥5 points, depression) [28]; (5) Mini Nutritional Assessment (MNA ≤ 8 points, malnutrition for nutritional status [29].

### 2.4. Frailty-Based Management of AS

We considered the associations between several factors, including clinical signs, surgical risk, nutritional markers, cognitive status, disability, comorbidity, and physical frailty score. Additionally, Fried criteria [21,30] were used to refer to tailored management of our elderly patients with AS. The sensibility and specificity of the score in this cohort of elderly patients were 0.84 and 0.82 (AUC = 0.763, 95% CI: 0.641–0.884), respectively, for Fried ≥ 3, distinguishing between pre-frail and early frail. 

No robust patients were found, pre-frail patients were directed to SAVR (Fried criteria = 1) and TAVR (Fried criteria = 2), while early frail patients (Fried criteria = 3) without acute heart failure (AHF) events or urgent interventions were scheduled for TAVR; early frail patients (Fried criteria = 3) with recent AHF events and creatinine > 1.4 mg/dL were scheduled for BAV, frail patients (Fried criteria = 4–5) for MT.

### 2.5. SAVR/TAVR/BAV Procedures and Follow-Up

Interventional procedures were performed within 60 days after the day service baseline examination, based on urgency. All patients underwent coronary angiography, providing information about the presence and treatment of coronary artery disease by percutaneous coronary intervention (PCI) or coronary bypass. SAVR was performed in extracorporeal circulation and minimally invasive sternotomy; biological valve aortic prostheses were used. The 38% of SAVR at angiography showed hemodynamic coronary artery disease and were treated with PCI/stenting. TAVR and BAV procedures were performed by trans-femoral (58% TAVR vs. 36% BAV) or secondary trans-radial access (42% TAVR vs. 64% BAV); trans-apical approach was not used. TAVR was performed under local anesthesia and mild conscious sedation, using Edward Sapien XT (Edwards Lifesciences, Irvine, CA, USA). and Medtronic Core Valve (Medtronic, Minneapolis, Minnesota) bioprosthesis. The median follow-up period was 20 months.

Patients referred to SAVR, TAVR, and BAV were checked for periprocedural complications, in particular, stroke, transient ischemic attack, myocardial infarction, life-threatening bleeding, acute kidney injury, coronary artery obstruction requiring intervention, major vascular complications, valve dysfunction requiring repeat procedure, or worsening chronic heart failure, which were recorded, as defined in the Valve Academic Research Consortium-2 consensus document [12].

### 2.6. Statistical Analysis

Continuous variables were reported as mean ± SD or median (IQR) depending on normality. Categoric variables were expressed as absolute numbers or percentages n (%). Normality was assessed using the Kolmogorov–Smirnov test. The comparison between groups was carried out by using ANOVA (with Bonferroni’s correction) for continuous data and the chi-square test for categorical variables. A two-sided *p*-value < 0.05 was considered statistically significant. The receiver operator characteristic (ROC) curve was constructed to assess the sensitivity and specificity of the Fried score. Kaplan–Meier and log-rank tests were considered to study survival for the three frailty levels in the treatment of the patients. Furthermore, univariate Cox regression analysis was performed to explore the association between the single covariates and the frailty level. Lastly, significant factors were tested again on multivariate Cox regression, with stepwise backward conditional elimination of non-significant factors in the model predicting the patients’ survival. Power of the study: we chose a medium effect size of 0.3. Considering this effect size, a study power (1-β) of 0.90 was expected with an α value of 0.05. The program, G*Power 3.1.2, was used for the calculation. All analyses were performed using IBM Statistical Package for Social Sciences (SPSS, version 23, Chicago, IL, USA, 2013).

## 3. Results

### 3.1. Clinical, Surgical, and Geriatric Data

One hundred and nine elderly patients with symptomatic severe AS undergoing a comprehensive pre-procedural evaluation were categorized into the following three groups based on the Fried frailty phenotype described in the Section 2: pre-frail (39.5%), early-frail (25.5%), and frail (35%). No patient resulted in a robust condition. Clinical, surgical, and geriatric features of all patients were compared with the Fried categories.

The clinical and surgical features are shown in Table 1: the mean age was 83.3 ± 5.5 years and 68% of patients were female. All patients had chronic heart failure with preserved ejection fraction (CHFpEF). The comorbidity rate was comparable in all groups. High levels of NYHA functional class, increasing STS score, systolic pulmonary artery pressure, decreasing EF, and moderate–severe mitral valve regurgitation were related to increased frailty. Patients with CHFpEF accounted for 51%, 68%, and 84% in the early, pre-frail, and frail groups, respectively, and patients with post-AHF events (occurred on average in the previous 52 days) with worsening CHFpEF increased from pre-frail to frail group (*p* = 0.0006).

An increasing trend in creatinine (*p* < 0.0001) and in BNP (*p* = 0.07) levels, going from the pre-frail to the frail group, was observed. Conversely, albumin values tended to decrease (*p* < 0.0001).

The prevalence of geriatric impairments according to physical frailty status is shown in Table 2: comorbidity increased with increasing physical frailty (CCI > 2, *p* < 0.0005), as well as malnutrition status (MNA ≤ 8, *p* < 0.0001), cognitive impairment (MMSE ≤ 18, *p* < 0.0001), and depression GDS ≥ 5, *p* = 0.023). Furthermore, disability was associated with physical frailty: lower IADL and BADL scores resulted in frail patients (*p* < 0.0001).

### 3.2. Frailty-Based Management of Elderly Patients with Symptomatic AS

According to Fried’s criteria, the patients received tailored treatment for AS. In particular, patients undergoing SAVR accounted for 19% of the total number of patients in the pre-frail group, whereas those undergoing TAVR accounted for 81% of those belonging to the pre-frail and 75% of those in the early group. Only 8% of the frail patients were addressed for TAVR because they successively needed urgent surgical treatment for neoplastic disease. A total of 25% of the patients treated with BAV belonged to the early group, while 55% belonged to the frail group. Additionally, 37% of patients treated with MT were part of the frail group (Table 1).

A small number of periprocedural complications were detected in pre-/early frail patients who underwent TAVR or BAV and these included vascular injury (18%/TAVR), bleeding (6%/TAVR), left bundle branch block/pacemaker implantation (8%/TAVR), STEMI (2%/BAV), and moderate paravalvular leakage (20%/BAV; 31%/TAVR). Eight patients, among those who underwent SAVR, were treated by “minimally invasive sternotomy”, and the complications were also evaluated by VARC-2: 25% of these patients showed only bleeding. About 10% of BAV patients additionally underwent a TAVR within twelve months.

### 3.3. Frailty and Mortality

**Figure 1:**
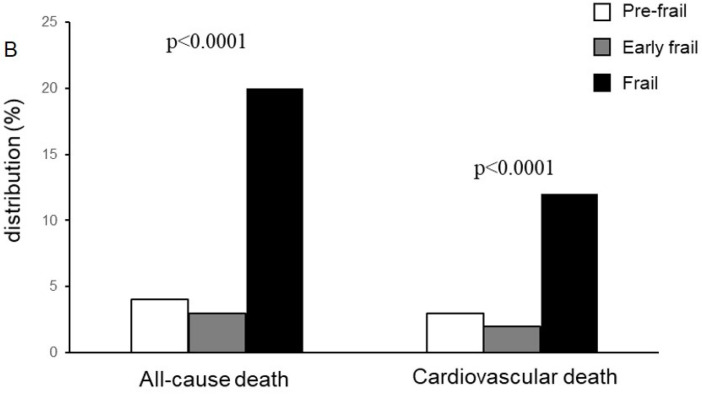
Distribution of all-cause mortality and CV death according to frailty status at 20 months. CV: cardiovascular.

At a median follow-up of twenty months, 25 patients (23%) died. The all-cause mortality resulted in a significant increase in the frail group (the frail group 52.3% vs. 7% and 7.3% in the pre-frail and early frail group, respectively; *p* < 0.0001) (Figure 1). In this group, 67% of the dead patients had been treated with MT, 45% with BAV, and 33.3% with TAVI. In the pre-frail group and early frail group, the dead patients had been treated with TAVR and BAV, respectively.

**Figure 2:**
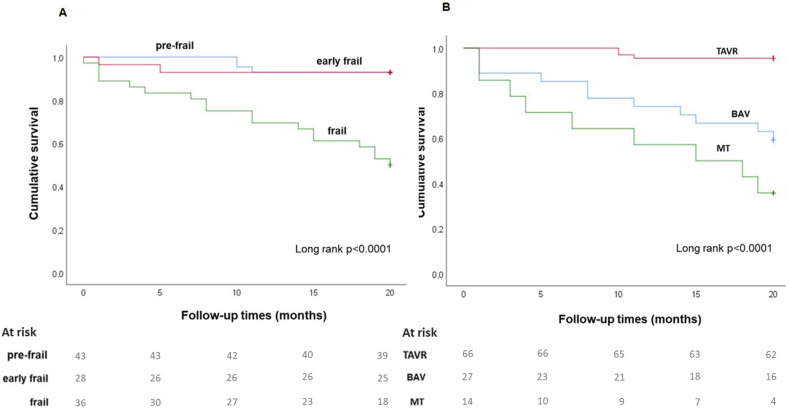
Kaplan–Meier survival curves. The survival rate at 20 months was higher in pre-frail patients ((**A**), long rank < 0.0001) and in the patients who underwent TAVR treatment ((**B**), long rank < 0.0001). Numbers of patients for frail group are shown at each time. Abbreviations as in Table 1.

Cardiovascular death was the first cause of mortality (76%) due to heart failure (HF) (65%), acute myocardial infarction (29%), and ictus (6%), followed by oncological disease (24%). Kaplan–Meier analysis showed that at 20-month follow-up, the survival rate was higher in the pre-frail patients (long rank < 0.0001, Figure 2A) and in the patients who underwent TAVR treatment (long rank < 0.0001, Figure 2B).

**Figure 3:**
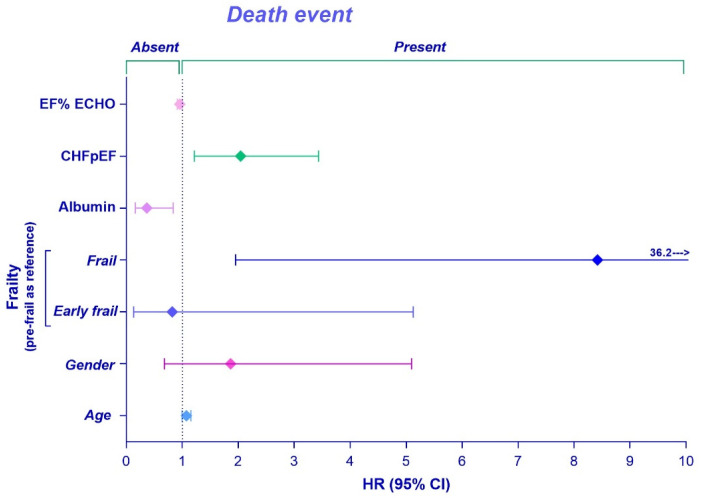
Cox proportional hazard regression analysis of risk of all-cause mortality in elderly AS patients. Frailty, CHF, albumin, and EF were associated with increased risk of all-cause death in a multivariate model adjusted for sex and age. HR: hazard ratio. Other abbreviations as in Table 1.

Univariate Cox regression analysis showed that CHFpEF, albumin, EF, and GDS were associated with an increased risk of all-cause of mortality. Multivariate Cox proportional hazard analysis (https://www.sciencedirect.com/topics/nursing-and-health-professions/proportional-hazards-model accessed on 6 March 2023) with stepwise backward conditional elimination of non-significant factors demonstrated that frailty (hazard ratio, HR (95% CI), 8.4 (1.95–36.25), *p* = 0.004), CHFpEF (HR (95% CI), 2 (1.21–3.43), *p* = 0.007), and EF (HR (95% CI), 0.95 (0.91–0.99), *p* = 0.043) were associated with an increased risk of all-cause death in a multivariate model adjusted for sex and age (Figure 3).

## 4. Discussion

In this study, for the first time, we used a pre-operative multidisciplinary pathway aimed at “frailty-based management” to identify a tailored treatment for severe AS in elderly patients. Through this approach, we highlighted how every single level of the frailty continuum must be well considered [31,32] to identify which patients may benefit from TAVR/SAVR, with respect to those in whom interventional or medical treatment is useless or palliative [7].

In fact, in older subjects, frailty syndrome is described as a dynamic process, ranging from the absence of frailty to pre-frail, early frail, and physically frail [21,31]. The pre-frail state increases the risk for progression to frailty, enhancing the risk for disability, vulnerability, adverse outcomes, and death [21,31,32,33]. Although frailty is considered a possible reversible process, Xue et al. measured the Fried frailty phenotype at different levels over time and its association with mortality, showing that all five frailty criteria are associated with an increased risk of mortality [32]. Moreover, high frailty scores were found to represent a critical point of irreversibility in the frailty continuum. The authors suggested that curative intervention in the early levels of frailty may be beneficial, whereas palliative care is indicated in advanced levels [32,33]. Frailty has also been associated with increased short- and long-term mortality after TAVR, using multiple scores in several clinical studies [14,15,16,17]. Furthermore, the evidence has demonstrated that the addition of frailty assessment in known prediction risk models improves the discrimination from short-term and mid-term mortality rates following TAVR [11,13,15]. Next to clinical and surgical risk, a multidimensional frailty approach, including the phenotype Fried score and MGA, was used in our study to choose a tailored therapeutic decision [34,35]. We found that the STS score, imaging, and laboratory parameters (NYHA class, EF, PAPs, and creatinine) and indices of malnutrition (MNA, sarcopenia, and albumin), comorbidity (CCI), disability (BADL and IADL), and cognitive (MMSE) and mood disorders (GDS) are strongly associated with the progression of the physical frailty score [21,31]. According to frailty-based management, using the Fried score, the pre-frail/early frail elderly patients that underwent TAVR/SAVR showed no significant periprocedural complications. Furthermore, no significant periprocedural complications or acute critical events occurred in early frail patients with recent acute heart failure events that underwent BAV as an emergent procedure. Hence, BAV still plays a useful role in improving symptoms and may be considered a bridge to definitive intervention. According to Bularga et al., BAV has a low procedural risk of mortality or stroke and can, thus, improve symptoms in some patients. Nevertheless, the mortality rate was very high in subjects who underwent a repeated BAV [36]. Additionally, advanced frail patients referred to MT were monitored by clinical follow-up.

After about 2 years of follow-up, all-cause mortality significantly prevails in frail and advanced frail patients on MT (67%), BAV (45%), and in TAVR-treated patients (8%) that needed urgent surgical treatment for neoplastic disease. This evidence suggests that BAV and MT must be carefully assessed by an expert heart team to avoid futile interventional treatment of AS in advanced frail patients, despite being palliative care [7]. Indeed, through multivariate COX analysis, frailty emerged as the most powerful independent factor associated with all-cause mortality, CHF, lower EF, and hypoalbuminemia. Frailty and HF share a common pathophysiological background, including inflammation, malnutrition, and sarcopenia [37], and are strongly associated with each other, thus justifying the use of comprehensive clinical and geriatric assessment and tailored therapy in elderly patients with AS and HF [38].

Finally, the novelty emerging from this study is that pre-frail/early frail elderly patients are the best candidates for aortic valve implantation by TAVR/SAVR. In these patients, characterized by low levels of comorbidity without significant disability, external blood interventions, such as SAVR/TAVR, result in beneficial outcomes because they improve early and late outcomes, as well as survival rates. Conversely, advanced frailty levels, comorbidity, disability, cognitive impairment, and malnutrition make patients more vulnerable to poorer outcomes after cardiovascular procedures; therefore, in these cases, palliative care is indicated [30,32,33]. Among palliative care, MT has been shown to prevent intervening with an aortic valve treatment. In fact, high Fried frailty scores are confirmed as a critical point of irreversibility [32,33] and are independently associated with an increased risk of mortality [4,5]. Often, elderly frail patients with severe AS are either asymptomatic or present masked NYHA class score symptoms due to the associated comorbidities [39]. Our data suggest that in elderly patients affected by degenerative aortic valve disease, better surveillance, making use of integrated frailty assessment, may be desirable during valve cardiologic controls for surgical scheduling. This clinical path, which quantifies the contribution of clinical, surgical, and geriatric risk factors, may identify a new “favorable time window” in which the interventional treatment of AS may be appropriate to interrupt the progression of valve disease, heart failure, and frailty syndrome [40] and reduce the risk of futility [18]. More and larger clinical studies using frailty markers linked with a higher mortality risk after aortic valve interventional procedures are required.

The major limitation of this work is that it is a single-center pilot study with a relatively small number of patients. However, we believe that our findings are strengthened by including an evaluation of clinical, surgical, and geriatric risk factors; selection based on Fried scores; and the relationship between the clinical phenotype and the prognosis. Moreover, as reported in the Section 2, a study power of 0.90 was expected (with a medium effect size of 0.3 and an α value of 0.05).

Nonetheless, in the multidimensional frailty assessment, we did not address the socio-economic component of frailty and did not evaluate the post-treatment frailty level and the quality of life of the patients in the clinical follow-up. Importantly, the assessment of the mortality rate in oncologic patients with MT or BAV might have affected the frailty assessment and survival. This aspect, although not included in the main aim of the present study, deserves to be better explored in future trials. However, in this regard, previous data have suggested that, whereas frailty is closely associated with cardiovascular mortality over a 5-year period in elderly subjects, no connection with death from cancer or other causes has been demonstrated [41,42].

## 5. Conclusions

Frailty has been confirmed to play a critical role in the decision making of elderly patients with severe AS. In particular, the application of a multidisciplinary pre-operative assessment, finalized to a “frailty-based management” of the patient and considering the *continuum* of a syndrome, highlighted that the pre-frail/early frail patients appear ideal candidates for TAVR/SAVR, with a high survival rate at middle term. Conversely, advanced frail patients with high comorbidity and disability are strongly subjected to mortality, independently of interventional or medical treatment (futile or palliative, respectively).

Thus, the evaluation of the frailty degree may be crucial to identify early and still-reversible stages of the syndrome, where AS interventional treatment may be implemented in favor of better clinical outcomes in the elderly population.

## Figures and Tables

**Table 1:** Baseline characteristics of study population according to frailty status.

Variable	All Patients (n = 109)	Pre-Frail (n = 43)	Early Frail (n = 28)	Frail (n = 38)	*p*-Value
Age (yrs)	83.3 ± 5.5	83.3 ± 4.7	84 ± 4.9	83 ± 6.6	0.72
Female, n (%)	74 (68)	26 (60)	17 (61)	31 (81)	0.08
*Comorbidities*					
Hypertension	97 (89)	38 (88)	25 (89)	34 (89)	0.98
Hypercholesterolemia	80 (73.4)	34 (79)	21 (75)	25 (65.7)	0.39
Diabetes	39 (35.8)	14 (32.5)	10 (35.7)	15 (39.4)	0.81
Smoking	29 (26.6)	16 (37.2)	4 (14.2)	9 (23.6)	0.06
COPD	46 (42.2)	20 (46.5)	11 (39.2)	15 (39.4)	0.76
Previous AMI	18 (16.5)	6 (14)	5 (17.8)	7 (18.4)	0.84
Previous stroke	15 (13.8)	2 (4.6)	7 (25)	6 (15.6)	0.08
CHFpEF	44 (40)	17 (39.5)	15 (53.5)	12 (31.5)	<0.0001
CLASSE NYHA					
I-II	70 (64.2)	35 (81.4)	18 (64.2)	17 (44.7)	0.002
III-IV	39 (35.8)	8 (18.6)	10 (35.7)	21 (55.2)	
Angina	33 (30.3)	14 (32.5)	8 (28.5)	11 (28.9)	0.91
PAPs	46.7 ± 11.3	43.5 ± 9.4	45 ± 9.5	51.7 ± 12.9	0.003
EF, %	57.4 ± 8.6	61 ± 6	58 ± 6.7	53.8 ± 10	0.0001
mAVG, mmHg	44 ± 12.3	45.6 ± 13	46 ± 9.3	40 ± 13	0.14
MVR (≥2+)	46 (42.2)	12 (28)	17 (60.7)	17 (44.7)	0.03
STS score	4.45 (2.7–6.1)	4 (2.5–4.6)	4.6 (4–5.5)	5.4 (2.3–9.8)	0.02
*Laboratory parameters*					
BNP, pg/mL	281 (134–588.2)	147.0 (105–346)	349.5 (134–628)	398.8 (223–774)	0.08
Creatinine, mg/dL	1.09 (0.9–1.4)	0.9 (0.85–1.13)	1.1 (0.85–1.5)	1.3 (1.1–1.9)	<0.0001
Albumin, g/L	4 ± 0.4	4.2 ± 0.3	4 ± 0.38	3.7 ± 0.5	<0.0001
*Aortic valve treatment*					<0.0001
SAVR	8 (7)	8 (19)	-	-	
TAVR	59 (54)	35 (81)	21 (75)	3 (8)	
BAV	28 (26)	-	7 (25)	21 (55)	
MT	14 (13)	-	-	14 (37)	

Values are mean ± SD, n (%), or median (interquartile range). BNP: brain natriuretic peptide; COPD: chronic obstructive pulmonary disease; AMI: acute myocardial infarction; CHF: chronic heart failure; NYHA: New York Heart Association; PAPs: systolic pulmonary artery pressure; EF: ejection fraction; mAVG: mean aortic valve gradient; MVR: mitral valve regurgitation; STS: Society of Thoracic Surgeons; SAVR: surgical aortic valve replacement; TAVR: transcatheter aortic valve replacement; BAV: balloon aortic valve; MT: medical therapy.

**Table 2:** Geriatric assessment according to frailty status.

Variable	All Patients (n = 109)	Pre-Frail (n = 43)	Early Frail (n = 28)	Frail (n = 38)	*p*-Value
Total number of drugs	6.7 ± 2.5	6.3 ± 2	7.2 ± 3.3	7.3 ± 2	0.07
Depressive symptoms, GDS pts Severe depressive symptoms (≥5 pts), n(%)	3.7 [1–5] 24 (22)	2 [1–3] 4 (9)	3.5 [1–5.5] 7 (25)	4 [2–9] 13 (34)	0.024
Charlson Comorbidity Index, CCI Comorbidities number > 2, n (%)	4.3 ± 2.2 87 (80.6)	3.3 ± 1.8 27 (62.7)	4.6 ± 2.4 23 (82)	5.2 ± 2.2 37 (97)	0.0005
Nutrition status, MNA pts Malnutrition (≤8 pts), n (%)	10.5 ± 2.3 19 (17)	11.8 ± 2.2 1 (2)	10.6 ± 2.2 3 (11)	8.6 ± 2.6 15 (39)	<0.0001
Cognitive status, MMSE pts Impaired cognition (≤18 pts), n (%)	24.5 (23–28) 15 (13.8)	27 (26–29) 10 (23)	25 (24–27) 14 (50)	21 (14–27) 23 (60)	<0.0001
Sarcopenia, n (%)	54 (49)	7 (16)	13 (46)	34 (89)	<0.0001
Disability					
BADL	5.1 ± 1.5	5.7 ± 0.96	5.4 ± 0.9	4.3 ± 1.9	<0.0001
IADL	6.1 ± 2.3	7.4 ± 1.3	6.6 ± 1.6	4.3 ± 2.4	<0.0001

Values are mean ± SD, n (%), or median (interquartile range). CCI: Charlson Comorbidity Index (score range, 1–12); GDS: Geriatric Depression Scale (score range, 0–13); MMSE: mini–mental state examination (score range, 1–30); MNA: Mini Nutritional Assessment (score range, 1–14); PASE: Physical Activity Scale for the Elderly; BADL: basic activities of daily living, (score range, 0–6); IADL: instrumental activities of daily living (0–8); pts: points of score.

## Data Availability

The dataset of the study is available from the corresponding author upon reasonable request.

## Abbreviations

AHF—acute heart failure; AMI—acute myocardial infarction; AS—aortic stenosis; AV—aortic valve; BADL—basic activities of daily living; BAV—balloon aortic valvuloplasty; BMI—body mass index; BNP—brain natriuretic peptide; CHFpEF—CHF with preserved ejection fraction; EF—ejection fraction; GDS—Geriatric Depression State; HR—hazard ratio; IADL—instrumental activities of daily living; LVEF—left ventricular ejection fraction; MMSE—mini–mental state examination; MNA—Mini Nutritional Assessment; MT—medical therapy; NYHA—New York Heart Association; PASE—Physical Activity Scale for the Elderly; PCI—percutaneous coronary intervention; SAVR—surgical aortic valve replacement; STS score—Society of Thoracic Surgeons; TAVR—transcatheter aortic valve implantation replacement; TUG—timed up-and-go; VARC—Valve Academic Research Consortium.
